# Long-term Chinese calligraphic handwriting reshapes the posterior cingulate cortex: A VBM study

**DOI:** 10.1371/journal.pone.0214917

**Published:** 2019-04-04

**Authors:** Wen Chen, Chuansheng Chen, Pin Yang, Suyu Bi, Jin Liu, Mingrui Xia, Qixiang Lin, Na Ma, Na Li, Yong He, Jiacai Zhang, Yiwen Wang, Wenjing Wang

**Affiliations:** 1 Advanced Innovation Center for Future Education, Beijing Normal University, Beijing, China; 2 State Key Laboratory of Cognitive Neuroscience and Learning, Beijing Normal University, Beijing, China; 3 IDG/McGovern Institute for Brain Research, Beijing Normal University, Beijing, China; 4 College of Information Science and Technology, Beijing Normal University, Beijing, China; 5 Department of Psychological Science, University of California, Irvine, California, United States of America; 6 Conservation Department, The National Palace Museum, Beijing, China; 7 School of International Journalism and Communication, Beijing Foreign Studies University, Beijing, China; 8 School of Arts and Media, Beijing Normal University, Beijing, China; Southwest University, CHINA

## Abstract

As a special kind of handwriting with a brush, Chinese calligraphic handwriting (CCH) requires a large amount of practice with high levels of concentration and emotion regulation. Previous studies have showed that long-term CCH training has positive effects physically (induced by handwriting activities) and psychologically (induced by the state of relaxation and concentration), the latter of which is similar to the effects of meditation. The aim of this study was to investigate the long-term CCH training effect on anxiety and attention, as well as brain structure. Participants were 32 individuals who had at least five years of CCH experience and 44 controls. Results showed that CCH training benefited individuals’ selective and divided attention but did not decrease their anxiety level. Moreover, the VBM analysis showed that long-term CCH training was mainly associated with smaller grey matter volumes (GMV) in the right precuneus/posterior cingulate cortex (PCC). No brain areas showed larger GMV in the CCH group than the control group. Using two sets of regions of interest (ROIs), one related to meditation and the other to handwriting, ROI analysis showed significant differences between the CCH and the control group only at the meditation-related ROIs, not at the handwriting-related ROIs. Finally, for the whole sample, the GMV of both the whole brain and the PCC were negatively correlated with selective attention and divided attention. The present study was cross-sectional and had a relatively small sample size, but its results suggested that CCH training might benefit attention and influence particular brain structure through mental processes such as meditation.

## 1. Introduction

As one of the most prominent elements of Chinese traditional culture, Chinese calligraphy can be traced back to more than 3000 years ago. In addition to its utility in conveying information, Chinese calligraphic handwriting (CCH) has developed special artistic value. Calligraphers use different scripting types (with seal, clerical, running, grass, and regular scripts as the five most popular scripts) to express ideas and create artworks. CCH is quite different from regular handwriting with a common pen or pencil. CCH is believed to integrate the mind and body into writing the Chinese characters [1]. It emphasizes the control of the brush, revealing strength without flaunting it and expressing inner psyche through the movements of the brush. Cognitively, CCH involves visual perception, spatial structuring, and planning [2].

Previous research has shown that CCH can have both physiological and psychological effects. Physiologically, CCH practice can lead to decelerated respiration, a slower heart rate, decreased blood pressure, and reduced muscular tension [3, 4], which are typical signs of relaxation. Psychologically, calligraphy has been found to heighten concentration and improve mood [5], reduce symptoms of mental illnesses [6, 7], improve cognitive abilities (e.g., perceptual abilities, working memory performance, attentional processes, [8–10], and change brain activity (as measured by EEG) [4, 11, 12] and functional connectivity among relevant brain areas (Chen et al., 2017). These effects are similar to those of relaxation and meditation training. Indeed, a previous study found that both CCH and meditation training reduced stress by showing increasing skin temperature and decreasing respiratory rate [13]. After all, CCH has been widely accepted as a spiritual practice involving self-cultivation, self-awareness and self-reflection in China [14].

Effective CCH practice requires accurate execution of strokes, which involves high concentration, emotion regulation, and self-awareness [6, 15, 16]. Three neural systems subserve the CCH writing: sensory feedback from the real-time graphic trace, bio-emotional feedback from the movement of arms and body, and cognitive feedback from the self-awareness of heighted attention, alertness, and quickened response during the CCH writing [15, 17]. The latter two types of processes involving attention, emotion regulation, and self-awareness are similar to those involved in mindful meditation [18]. Therefore, the current study focused on the key ROIs that are involved in attention control, emotion regulation, and altered self-awareness, the three vital components of mindfulness meditation as articulated by Tang et al. (2015)[18].

We hypothesized that long-term CCH training would affect the brain in the same way meditation does. Thus far, to the best of our knowledge, no study has investigated CCH training and brain structure. In this study, we investigated the effect of long-term CCH training effect on grey matter volume (GMV) throughout the whole brain with a voxel-based morphometry (VBM) method. In addition, we explored whether CCH training mainly reshaped the brain structure related to handwriting or meditation. We focused on five brain areas that have been identified as key areas involved in handwriting [19, 20], including superior parietal lobule/anterior part of the intraparietal sulcus, SPL/IPS; graphemic/motor frontal area, GMFA; ventral premotor area, vPM; posterior cerebellum, postCB; and six brain areas that have been associated with meditation [18] (anterior cingulate cortex, ACC; frontopolar cortex, PFC; posterior cingulate cortex, PCC; Insula; striatum; amygdala). Finally, we correlated indices of brain structure with behavioral measures of attention and anxiety.

## 2. Materials and methods

### 2.1. Participants

The sample of the present study came from a larger project as reported in Chen et al. (2017) [10], with 86 participants (n = 36 for the CCH group; n = 50 for the control group). Of the total sample, 76 had brain imaging data, which were used in the present study. The CCH group had 32 participants who majored in CCH with at least five years of formal training and the control group had 44 participants who majored in social sciences and humanities other than CCH and had no more than a few months of basic CCH skill training. All participants were recruited from Beijing Normal University, Beijing, China. They were all right-handed native Chinese speakers. This study was approved by the Institutional Review Board of the State Key Laboratory of Cognitive Neuroscience and Learning at Beijing Normal University, China. All participants signed an informed consent form after a full explanation of the study procedure and were compensated for their time.

### 2.2 Major behavioral measures

#### 2.2.1 Raven’s advanced progressive matrices (APM) test

All participants completed both Set I and Set II of the Raven’s APM test [21]. The standard instructions were read aloud to the subjects, and the time limits were 5 minutes and 40 minutes for Set I and Set II, respectively. The scores from Set II were used to index IQ.

#### 2.2.2 The anxiety subscale of the symptoms checklist-90 (SCL-90)

SCL-90 is a widely used scale to assess mental health status. The anxiety subscale of the SCL_90 was used in this study to assess general anxiety symptoms, which are supposed to be affected by long-term CCH training. This subscale consists of 10 items scored on a 5-point Likert scale (from 0 to 4) and the range of the total score is 0−40. Following the examples of several recent studies that use SCL-90/SCL-90-R as a continuous measure (e.g., to investigate gender differences in psychological distress, anxiety and depression among late adolescents [22], or to compare psychological states of young adult adoptees and the general population [23]), we used the continuous sum score in the current study.

#### 2.2.3 The state-trait anxiety inventory (STAI)

The STAI is a standardized 40-item questionnaire used to evaluate both state and trait anxiety. State anxiety refers to fear, nervousness, discomfort, and arousal of the autonomic nervous system induced from the temporary environment, and trait anxiety means a relatively enduring disposition to feel stress, worry, and discomfort [24]. The Chinese version of STAI [25] was used in the present study.

#### 2.2.4 Selective attention measurement

We used the cue-target task to measure selective attention. The computerized version of this task included 10 practice trials and 120 formal trials with three stimulus onset asynchrony (SOA) of 50 ms, 250 ms, or 950 ms that separated the cue and the target. Participants responded to the peripheral target while remaining visually fixated at the center of the screen. The targets were preceded by a visual cue, which might occur in the same location as the subsequent target (valid trials) or in a location contralateral to the target (invalid trials) or without cue (no-cue trials). The reaction time (RT) and accuracy were recorded. Two indices were created for each SOA condition (50 ms, 250 ms, and 950 ms): the indices of Benefit (B_50, B_250 and B_950) were calculated by subtracting the valid cue RTs from the no-cue RTs and the indices of Cost (C_50, C_250 and C_950 were calculated by subtracting the no-cue RTs from the invalid cue RTs) [26].

#### 2.2.5 Divided attention measurement

A dual task paradigm was used to measure divided attention. The task included 14 practice trials and 96 formal trials. For every trial, a letter (S, D, F) and a number (1–6) were presented at the same time in the horizontal direction, with the letter on the left side and the number on the right side. Participants responded by typing the presented letter (S, D, or F) with the left hand, and typing the ‘5’ with the right hand if “5” was presented but otherwise ignoring any other numbers. At the very beginning and between trials, a red cross was placed at the center of the computer screen. The reaction time (RT) and accuracy were recorded, and the hit rate (HR) and false alarm rate (FAR) were calculated.

### 2.3 Brain imaging data collection and preprocessing

#### 2.3.1 fMRI data acquisition

All scanning was performed using a SIEMENS TRIO 3-Tesla scanner in the Brain Imaging Center of Beijing Normal University. Participants were told not to engage in heavy physical activities or to have stimulating drinks the day before the scanning. Each participant underwent a 3D anatomic session and an eight-minute resting-state fMRI (RS-fMRI) scanning session. The 3D T1-weighted magnetization- prepared rapid gradient echo (MPRAGE) image was acquired with the following parameters: 144 sagital slices, slice thickness/gap = 1.3/0.65 mm, TR = 2530 ms, TE = 3.39 ms, inversion time (Ti) = 1100 ms, flip angle = 7°, FOV = 256×256 mm2, matrix size = 256×192. During the RS-fMRI session, the participants were instructed to keep their eyes closed, stay as still as possible, and not to think about anything in particular. Images were obtained with the following parameters: 33 axial slices, thickness/gap = 3.5/0.7 mm, matrix size = 64×64, repetition time (TR) = 2000 ms, echo time (TE) = 30 ms, flip angle = 90°, field of view (FOV) = 200×200 mm^2^.

#### 2.3.2 Image preprocessing and analysis

**2.3.2.1 Voxel-based morphometry (VBM) analysis.** This study used VBM analysis to investigate brain anatomy. Structural brain images were processed using the VBM8 toolbox (http://dbm.neuro.uni-jena.de/vbm.html) and the SPM8 software. Data preprocessing was completed using default parameter setting for the following five main steps: 1) Reorienting the T1 images of all participants with the default reference coordinates; 2) segmenting the structural images into gray matter, white matter and cerebrospinal fluid; 3) Normalizing gray and white matter to Montreal Neurological Institute (MNI) standard space with the high dimensional DARTEL method; 4) modulating the images using the non-linear components approach to correct the differences of individual brains; and 5) smoothing with FWHM = 8mm using SPM8. Statistical analyses were also performed in SPM8. Group analyses were conducted for both the whole brain and regions of interest (ROIs) with age, gender, IQ, and total volume of brain tissue as covariates. Voxels with grey value < .1 were eliminated. In the whole brain analysis, a two-sample T-test was performed with a topological false discovery rate (FDR) correction (p< .001) [27–31].

For ROI analyses, we used five brain areas involved in handwriting [19, 20], including IPS/SPL (MRIcro number 57,58), GMFA (3,4), vPM (1,2), VWFA (55,56) and postCB (112), and six brain regions related to meditation [18], including ACC (31,32), PFC (3,4,7,8,23–26), PCC (35,36), insula (29,30), striatum (71–74), amygdala (41,42) (S1 Table). All of the eleven ROIs were extracted from AAL template using Slice Viewer in REST 1.8(http://restfmri.net/forum/REST_V1.8). A more stringent multiple comparison correction method (family wise error, FWE, p< .05) was used for the ROI group analyses. The FWE correction was applied at the voxel level of each ROIs. GMV of ROIs that showed significant group difference were extracted for correlational analyses.

**2.3.2.2 Correlation analysis.** We used partial correlation analyses to investigate the relationship between GMV (the whole brain and PCC) and behavioral performance on the cue-target paradigm task (including RT, HR and FAR) and the dual task paradigm (including the B_50, B_250, B_950, C_50, C_250 and C_950), with age, gender, and IQ as covariates.

## 4. Results

### 4.1 Demographic data

The CCH and control groups did not differ in terms of gender (χ2 = 0.627, p = 0.428), age (t = -0.697, p = 0.488), years of education (t = -0.551, p = 0.583), and IQ (t = -1.277, p = 0.206). The CCH practitioners had 5–20 years of experience (M = 10.69 years, SD = 3.55); they started practicing at 5–20 years of age (M = 9.14 years, SD = 4.05), and practiced CCH on average for 0.50–7.00 hours per day (M = 2.44 years, SD = 1.38) (Table 1). In terms of the type of scripts, most participants had experience with at least two or three types of scripts, with the regular and the running scripts being the most common.

**Table 1:** Sample characteristics of the CCH and the control groups.

Variables	CCH	Controls	*t or χ2*	*p*
N (Male/Female)	32 (13/19)	44(14/30)	0.627	0.428
Age (mean±SD in year)	21.23±2.11 (18.08~26.42)	21.61±2.54 (17.17~28.42)	-0.697	0.488
Handedness (% of right hand)	100	100		
Education (mean ±SD in year)	14.34±2.04 (9~19 )	14.59±1.85 (12~18)	-0.551	0.583
IQ	26.47±3.68 (18~33 )	27.58±3.74 (21~35)	-1.277	0.206
Years of practicing CCH (mean±SD in year)	10.69±3.55 (5~20)			
The age of starting practicing CCH (mean±SD in year)	9.14±4.05 (5~20)			
Mean hours of practicing CCH per day (mean±SD in hours)	2.44±1.38 (0.50~7.00)			

### 4.2 Behavioral results

In terms of anxiety, we did not find significant differences between the CCH and the control groups with the SCL_90 anxiety subscale (t = 0.116, p = 0.416), the STAI state anxiety (t = -0.73, p = 0.942), or the STAI trait anxiety (t = 0.139, p = 0.890).

For both the cue target and divided attention tasks, non-valid trials (i.e., negative validity effect trials and outliers beyond three standard deviations) were deleted. In terms of attention, long-term CCH training seemed to have positive effects on both the divided attention and selective attention (Data are available in S2 Table). In the cue-target task, the CCH participants showed a shorter RT in valid cue trials with SOA of 50ms (t = -2.675, p = 0.009) and 250ms (t = -2.159, p = 0.034) than control subjects, but not for the condition with the SOA of 950ms. The two groups did not differ in accuracy. Nor did they differ in RT for the invalid and no-cued trials. On the dual task, the CCH group had a significantly higher hit rate (t = 2.777, p = 0.007) and a lower false alarm rate (t = -2.879, p = 0.005) than the control group.

### 4.3 Results of VBM

#### 4.3.1 Whole brain analysis

**Fig 1:**
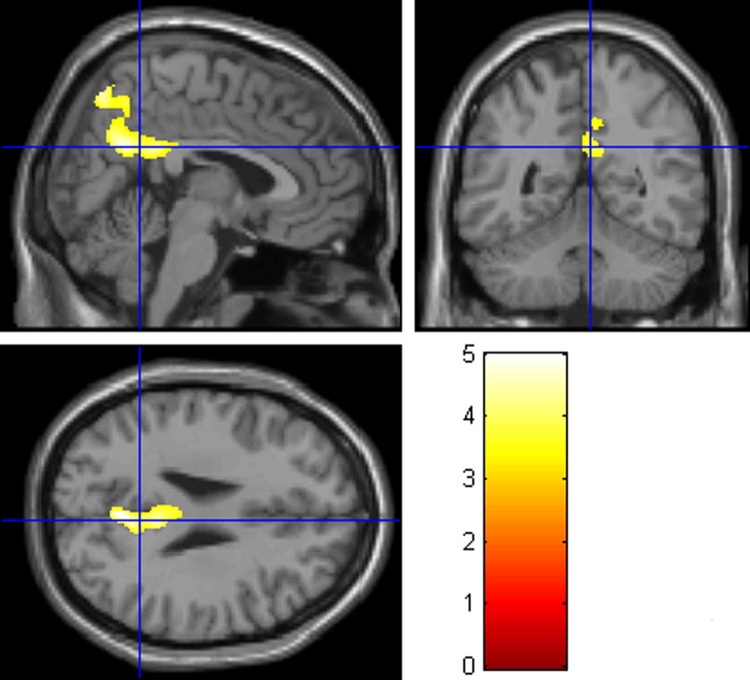
Brain areas that showed smaller GMV in the CCH group than the control group with topological FDR (p< .01). The coordinates of the cross are [5, –51, 27].

Whole-brain analysis found one significant cluster (voxel size = 1573) located in the right precuneus, including the bilateral precuneus, right middle cingulate gyrus and bilateral posterior cingulate cortex, right superior parietal lobule (Fig 1 and Table 2). The CCH group had smaller GMV than did the control group. No brain areas of the CCH group were found to have larger GMV than the control group.

**Table 2:** Clusters that showed smaller GMV in the CCH group than the control group based on whole brain analysis (FDR correction, p< .001) and ROI analysis (FWE correction, p< .05).

Cluster size	Peak(MNI)			Side	Cluster location	Brodmann areas (BA)	Peak T
	X	Y	Z				
*Whole brain analysis*							
1573	3	-60	28	R	precuneus	23	4.99
	6	-70	57	R	precuneus	7	4.48
	5	-42	27	R	PCC	26	4.32
*ROI analysis*							
390	5	-42	27	R	PCC	26	4.34
	2	-52	28	R	PCC	23	3.49
	2	-33	30	R	PCC	23	3.45

#### 4.3.2 ROI analysis

**Fig 2:**
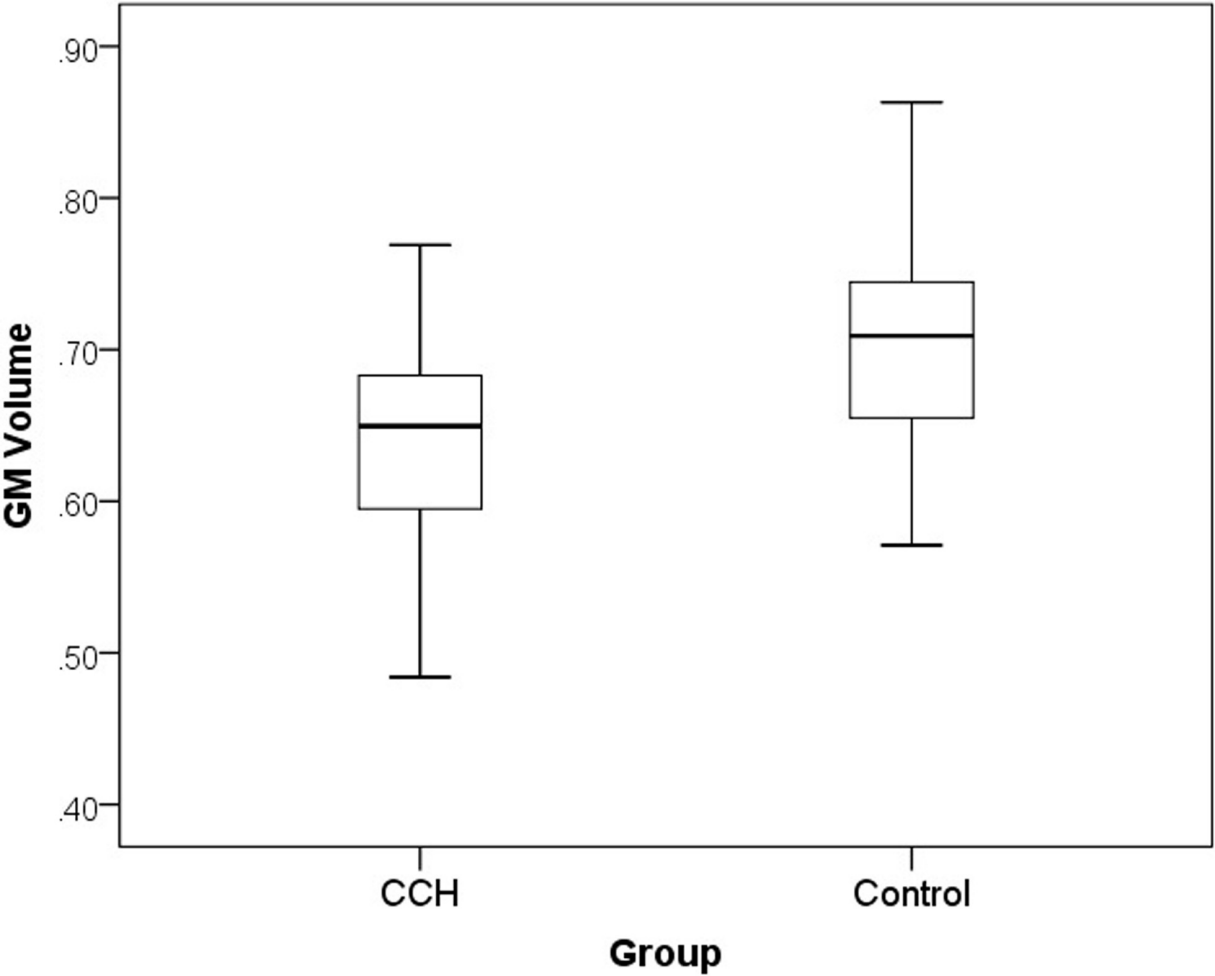
Box plot of grey matter volumes across the whole brain of CCH participants and controls. The central box represents the value from the lower to upper quartile (25th to 75th percentile). The middle line represents the median. The vertical line extends from the minimum to the maximum value.

**Fig 3:**
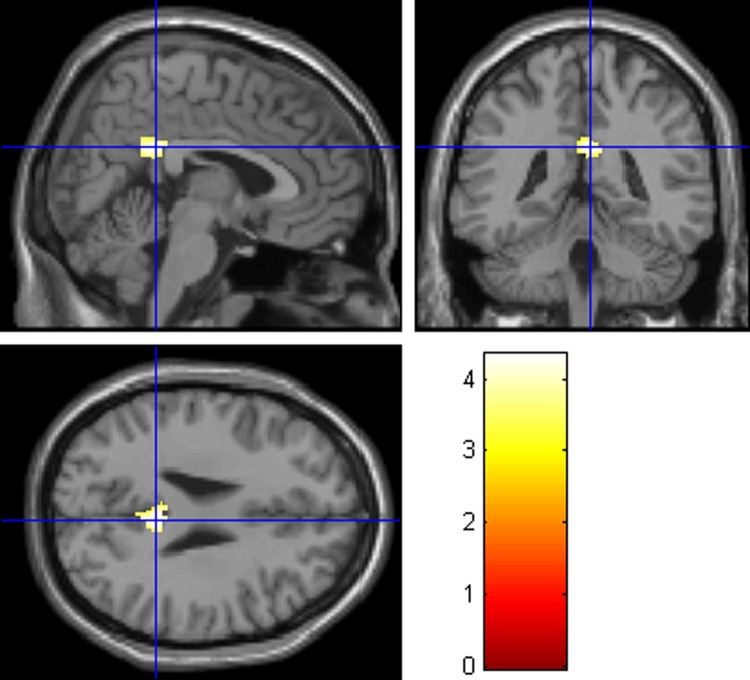
Brain area that showed smaller GMV within the PCC in the CCH group than the control group, with FWE correction (p< .05). The coordinates of the cross are [5, –42, 27].

Using the ROIs related to either handwriting or meditation, ROI analysis showed that only the GMV of PCC differed between the two groups of participants (Table 1 and Figs 2 and 3), confirming the whole-brain analysis. No group difference of the other ROIs survived the FWE (p< .05) correction.

### 4.4 Correlation results

**Fig 4:**
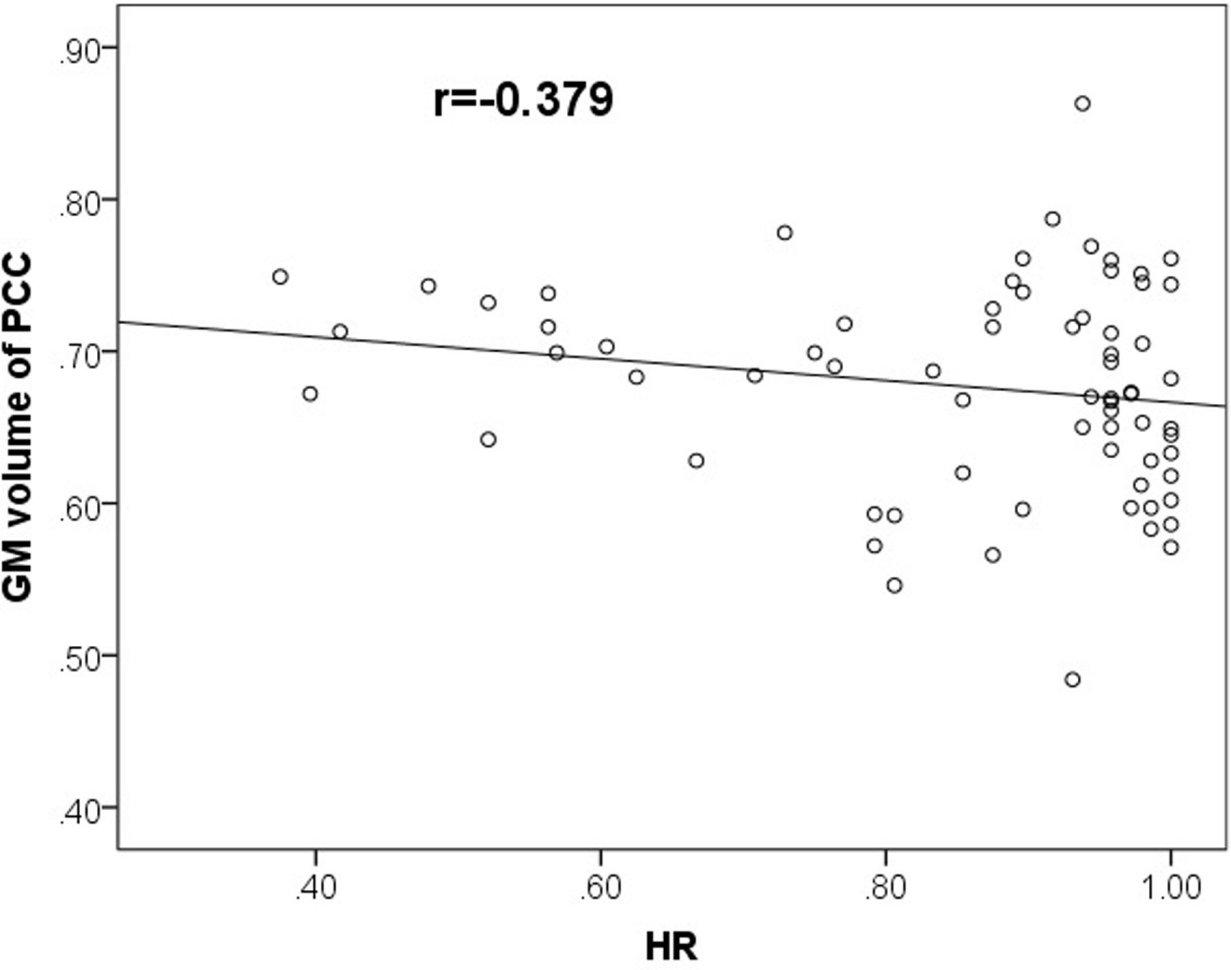
Gray matter volume (GMV) of the PCC was negatively correlated with the HR of the dual task.

Across all participants, GMV of the whole brain was negatively correlated with the B_50 of the cue-target task (r = -0.333, p = 0.018). Because only the GMV of PCC showed a significant group effect, we further correlated it with behavioral measures. Results showed that the GMV of PCC had a significant negative correlation with the HR of the dual task (r = -0.379, p = 0.007) (Fig 4). Moreover, even though we did not find a group difference for the mean RT of the dual task, correlation analysis revealed that it was significantly and positively correlated with the GMV of PCC (r = 0.304, p = 0.032).

## 5. Discussion

The current study explored the effect the long-term experience with CCH on both behavioral outcomes (anxiety and attention) and brain structure (GMV). Results indicated that individuals with at least five years of CCH experience showed better performance on the selective and divided attention tasks. VBM results showed that long-term CCH training could lead to decreased GMV (mainly in the precuneus/PCC). Correlation analysis revealed that the GMV of the whole brain and PCC had negative relations with selective and divided attention, respectively.

Previous studies have found that CCH intervention could help patients to relax and improve their mood [32], to reduce stress [13], and to help ADHD children to focus their attention (Kao, 2006). Our results confirmed the benefits of CCH training on attention, but not on anxiety (either measured with the anxiety subscale of SCL_90 or STAI questionnaire). One explanation of this discrepancy is that our participants were healthy young adults, whereas previous studies’ participants were patients. Perhaps the positive effects of CCH practice on psychological level are not as evident among healthy individuals as in patients.

In terms of the cue-target task, we found the benefits of CCH training only for two stimulus onset asynchronies (SOA) (50 ms and 250 ms), but not for the 950 ms. It might be due to the fact that the inhibitory aftereffect counteracted the benefit of CCH training to top-down attention. Inhibitory aftereffect (later labeled as ‘inhibition of return’, IOR) has been described since 1984. For the cue-target task, it refers to the phenomenon that RTs are facilitated to targets that appeared at the same locations as the cue at short SOA, but the RTs are inhibited as the SOA exceeds 300 ms. The facilitation is supposed to be due to a reflexive shift of attention towards the source of stimulation [33], which induces an expectation-based process (top-down). IOR is attributed to orienting attention away from the cue location [34]. Because this study found an effect of CCH training only for SOAs of 50ms and 250ms, we speculate that CCH training mainly benefits the top-down attention control ability.

The results of VBM showed that the CCH group had significantly smaller GMV than the control group in bilateral precuneus, right middle cingulate gyrus and bilateral PCC, and right superior parietal lobule (rSPL). These areas have been associated with meditation or mindfulness [18, 35, 36], but PCC is most consistently linked to meditation [37]. Furthermore, our finding of negative associations was consistent with the previous findings that trait mindfulness was correlated with smaller GMV in PCC [38] and that meditators had smaller cortical thickness than controls [39]. PCC has long been thought to be involved in attention processing [40], and studies have shown that PCC was deactivated during focused attention and meditation [41, 42]. Moreover, we found that selective attention performance was correlated negatively with the whole brain GMV. This negative correlation was consistent with that between the divided attention performance and GMV in PCC. These results suggested that both selective attention and divided attention were facilitated by increasing the efficiency of brain networks [10], albeit through different brain networks for the two kinds of attention. Indeed, we previously reported that the CCH group showed stronger resting-state functional connectivity (RSFC) than the control group in PCC and other brain areas [10].

It is worth noting that most of the brain areas showing smaller GMV in the CCH group than the control group were on the right hemisphere (83.15% voxels), which has been found to be particularly relevant to the processing of ideographic characters as compared to alphabetic writing [43, 44]. We used ‘Chinese character/ideographic character’ as key words in the Neurosynth system, and found 22 studies. Focusing on the general brain mechanisms of processing Chinese characters, six studies found that PCC/precuneus was activated [45–50]. Besides, among the three studies investigating the neural mechanisms of writing Chinese characters, PCC/precuneus was also found involved [49]. However, due to the particular characteristics of CCH training as mentioned in the Introduction section, we speculated that CCH training mainly facilitated the attention process through meditation-related mechanisms rather than visual spatial processes in PCC. Indeed, this study found that CCH training benefitted attention based on behavioral measures and a recently study also found that the main brain activity difference was in PCC between meditators and nonmeditators when performing an attention task [51].

It is also worth noting that the effects we found could not be attributed to mere exposure to Chinese characters. If exposure had been a factor, the usage frequency effect would be found in the same brain regions. However, the frequency effects of Chinese character processing have been localized to the left hemisphere [52, 53], rather than the right atmosphere as found in the current study. Moreover, as written language is ubiquitous in our daily life, it is hard to imagine that the two groups of participants would have different amounts of exposure to Chinese characters overall. Therefore, we would attribute our findings to the unique characteristics of CCH training, which, as mentioned earlier, involves not just brush control but also mental control.

We did not find group differences in the GMV of ROIs related to handwriting. One explanation is that the control participants also engaged in much handwriting, albeit with hard-tipped pens rather than brushes.

There were several limitations of this study. First, the cross-sectional nature of this study does not allow us to draw causal conclusions. A longitudinal or training design is needed to address the question of causality. Second, we relied on previous research to identify meditation- and handwriting-related ROIs. Future research should include a meditation group. Third, this study had a relatively small sample size.

## Supporting information

S1 Table Five brain areas involved in handwriting and six areas involved in meditation. (PDF)

S2 Table Behavioral data of cue-target task and dual task paradigm. (PDF)
