# Supplementary material for: Long-term Chinese calligraphic handwriting reshapes the posterior cingulate cortex: A VBM study
Source: PLoS One. 2019 Apr 4;14(4):e0214917. doi: 10.1371/journal.pone.0214917 (PMC6448813; doi:10.1371/journal.pone.0214917)
Supplement: S1 Table — (PDF) [file pone.0214917.s001.pdf]

Long-term Chinese calligraphic handwriting reshapes the posterior cingulate cortex: a VBM study

Supplemental Materials

S1 Table. Five brain areas involved in handwriting and six areas involved in meditation.

Behavioral dimensions	ROIs	AAL Brain Areas	Mricro Number
Handwriting	IPS/SPL	Postcentral_L<br>Postcentral_R	57,58
	GMFA	Frontal_Sup_L<br>Frontal_Sup_R	3,4
	vPM	Precentral_L<br>Precentral_R	1,2
	VMFA	Fusiform_L<br>Fusiform_R	55,56
	postCB	Vermis_6	112
Meditation	ACC	Cingulum_Ant_L<br>Cingulum_Ant_R	31,32
	PFC	Frontal_Sup_L<br>Frontal_Sup_R<br>Frontal_Mid_L<br>Frontal_Mid_R<br>Frontal_Sup_Medial_L<br>Frontal_Sup_Medial_R<br>Frontal_Mid_Orb_L<br>Frontal_Mid_Orb_R	3,4,7,8,23,24,25,26
	PCC	Cingulum_Post_L<br>Cingulum_Post_R	35,36
	Insula	Insula_L<br>Insula_R	29,30
	Striatum	Caudate_L<br>Caudate_R<br>Putamen_L<br>Putamen_R	71,72,73,74
	Amygfala	Amygdala_L<br>Amygdala_R	41,42
