# Supplementary material for: Long-term Chinese calligraphic handwriting reshapes the posterior cingulate cortex: A VBM study
Source: PLoS One. 2019 Apr 4;14(4):e0214917. doi: 10.1371/journal.pone.0214917 (PMC6448813; doi:10.1371/journal.pone.0214917)
Supplement: S2 Table — (PDF) [file pone.0214917.s002.pdf]

Long-term Chinese calligraphic handwriting reshapes the posterior cingulate cortex: a VBM study

Supplemental Materials

S2 Table. Behavioral data of cue-target task and dual task paradigm.

ID	Cue-target task									Dual task paradigm		
	RT (ms)											
	valid_50	invalid_50	uncued_50	valid_250	invalid_250	uncued_250	valid_950	invalid_950	uncued_950	RT(ms)	HR	FAR
CCH001	317.88	350.58	382.00	324.29	349.92	434.50	342.17	317.33	332.50	648.25	1.00	0.00
CCH002	393.88	428.67	430.50	410.04	451.50	447.50	341.29	349.00	379.50	913.30	0.97	0.03
CCH003	367.63	399.00	477.00	332.33	352.25	360.00	338.96	315.67	374.00	870.34	0.96	0.00
CCH004	389.13	403.00	414.50	357.96	384.83	437.50	350.13	358.42	362.00	1008.85	0.96	0.00
CCH005	338.33	404.25	398.00	348.92	398.92	447.00	331.83	306.58	315.50	952.88	0.94	0.00
CCH006	390.79	415.25	419.00	339.96	391.75	406.50	386.92	379.33	421.50	736.91	0.99	0.00
CCH007	305.38	350.75	421.50	308.54	343.17	401.50	316.92	302.83	298.00	698.37	1.00	0.00
CCH008	364.42	375.25	538.50	379.63	379.58	477.00	376.63	352.17	377.50	1076.68	0.83	0.01
CCH009	354.50	344.17	544.00	358.38	351.75	414.50	342.88	336.42	457.00	788.01	0.93	0.01
CCH010	308.25	313.25	440.00	306.38	301.33	279.50	339.79	276.08	300.50	795.70	0.96	0.01
CCH011	411.83	410.33	518.50	352.38	357.75	435.00	371.83	346.25	470.00	745.94	1.00	0.00
CCH012	314.29	357.67	423.50	305.71	375.58	458.00	370.96	349.92	348.50	879.06	0.94	0.03
CCH013	376.71	410.42	401.00	374.25	385.33	360.50	411.42	384.42	378.00	891.82	0.93	0.03
CCH014	361.75	380.17	435.00	312.46	322.50	396.00	329.50	317.83	376.50	790.00	1.00	0.00
CCH015	349.63	394.17	591.50	321.79	374.92	420.00	388.08	358.25	423.50	818.38	0.96	0.06
CCH016	323.83	342.58	388.00	311.13	316.42	339.00	339.79	308.42	405.00	734.08	0.97	0.03
CCH017	361.42	375.75	367.00	324.21	351.33	333.00	344.96	321.17	415.00	856.92	0.79	0.03
CCH018	352.96	361.08	415.50	323.42	337.58	340.00	366.29	350.50	489.50	824.75	0.71	0.00
CCH019	417.46	448.58	632.50	388.42	425.33	504.00	382.58	383.42	467.50	868.26	0.79	0.03
CCH020	351.04	394.00	403.00	351.42	350.92	384.50	329.58	336.33	400.50	710.60	0.81	0.03
CCH021	391.00	424.42	710.00	382.25	408.58	460.00	394.71	417.33	377.50	1060.88	0.76	0.01

CCH022	312.83	327.42	385.00	298.04	296.92	322.00	319.13	311.08	318.00	742.65	0.81	0.08
CCH023	339.83	376.83	380.00	329.63	363.50	381.50	371.42	336.67	439.50	803.96	0.89	0.03
CCH024	302.71	340.50	567.50	291.96	335.50	363.00	324.42	322.92	303.50	824.17	0.57	0.01
CCH025	312.29	335.17	371.50	294.42	362.33	379.00	286.67	297.58	314.00	616.71	0.99	0.03
CCH026	332.75	348.42	369.00	326.42	342.83	398.50	341.33	323.75	325.00	772.04	0.97	0.00
CCH027	420.33	465.92	518.00	334.17	383.92	477.00	476.75	432.92	443.00	883.61	1.00	0.03
CCH028	305.33	335.08	390.50	281.38	312.58	235.33	347.42	337.17	325.50	992.49	0.99	0.01
CCH029	327.21	362.17	360.50	329.38	387.75	365.50	344.00	335.67	461.00	950.26	0.96	0.00
CCH030	324.58	376.17	407.00	319.50	299.08	355.00	321.29	279.58	338.00	996.92	0.98	0.08
CCH031	306.92	319.92	303.00	300.54	309.08	289.50	320.46	303.33	372.00	919.32	0.88	0.06
CCH032	330.79	348.50	407.00	322.67	353.17	402.50	364.63	342.58	352.50	744.30	1.00	0.00
CON001	318.38	327.17	317.00	310.00	353.42	343.00	320.13	290.42	311.00	886.55	0.40	0.19
CON002	325.64	365.10	331.75	280.95	393.46	341.00	284.42	417.91	313.00	931.60	0.73	0.23
CON003	344.61	364.89	325.00	315.75	334.00	313.00	328.27	334.33	299.00	736.96	0.96	0.02
CON004	322.17	349.75	369.08	307.88	376.16	361.50	318.50	315.17	351.00	811.82	0.96	0.04
CON005	331.17	274.00	346.08	338.62	263.73	301.00	329.31	289.64	360.00	805.34	0.77	0.04
CON006	347.30	402.89	395.00	334.18	328.36	563.67	350.56	286.93	391.00	1248.48	0.63	0.35
CON007	410.32	383.63	490.50	386.67	350.00	477.00	364.04	331.57	402.00	886.17	0.90	0.00
CON008	352.83	347.83	372.50	308.83	318.17	406.50	367.42	326.25	365.50			
CON009	417.08	436.50	450.67	395.50	395.50	429.00	391.50	374.58	526.50			
CON010	304.33	343.31	372.58	305.00	319.45	337.00	275.13	285.44	306.00	904.59	0.52	0.08
CON011	337.76	402.54	329.00	310.96	412.33	557.00	323.77	356.70	542.00	931.67	0.85	0.06
CON012	343.35	383.88	488.67	355.38	336.67	663.50	345.54	329.33	566.00	787.72	0.88	0.17
CON013	301.54	388.69	529.58	278.59	363.64	398.00	294.57	334.89	244.00	995.87	0.48	0.04
CON014	339.95	380.60	339.00	324.05	342.80	331.00	286.65	334.69	306.00	1011.77	0.56	0.13
CON015	525.55	341.43	387.50	441.44	326.36	429.00	462.90	300.00	523.00	806.31	0.94	0.06

CON016	308.61	526.71	306.58	284.42	447.22	330.00	341.82	409.93	298.00	1015.54	0.90	0.04
CON017	293.10	366.00	442.50	294.23	388.56	220.67	311.32	284.70	305.67	764.54	0.42	0.06
CON018	313.67	330.58	339.25	272.16	359.18	282.00	307.71	439.89	315.00	674.53	0.56	0.00
CON019	440.75	491.58	594.00	425.17	416.42	569.00	456.83	405.50	412.00			
CON020	467.69	313.00	401.50	410.24	342.21	481.00	375.77	308.92	377.50	1018.33	0.88	0.04
CON021	513.46	575.83	689.42	516.75	674.25	551.50	498.46	483.50	482.00	912.72	0.90	0.04
CON022	419.14	492.00	643.00	433.76	376.64	311.50	402.38	284.50	391.00	899.83	0.60	0.13
CON023	383.00	360.50	509.50	346.71	345.58	324.00	354.88	352.80	313.00	936.53	0.52	0.06
CON024	390.00	384.09	552.00	381.35	314.09	594.50	325.73	317.40	517.00	947.33	0.67	0.15
CON025	368.00	348.55	365.50	332.83	359.75	317.00	342.46	317.92	295.50			
CON026	362.63	380.83	406.58	317.46	347.50	377.50	350.04	345.00	683.50	844.86	0.38	0.02
CON027	378.73	414.14	445.83	362.75	323.56	353.00	377.55	336.07	320.50	1002.99	0.75	0.13
CON028	445.08	303.25	355.00	392.33	288.67	473.50	380.08	347.20	684.50	685.57	0.98	0.04
CON029	479.75	541.33	530.00	458.17	503.83	523.50	465.46	466.83	442.50	686.99	0.96	0.00
CON030	429.00	440.58	545.50	382.79	386.67	446.50	421.67	402.33	462.00	670.07	1.00	0.00
CON031										940.67	0.94	0.00
CON032	402.18	451.83	448.00	357.38	391.50	407.00	375.17	322.83	353.50	1026.79	0.92	0.04
CON033	386.38	428.00	457.50	384.08	416.75	425.00	404.13	386.67	396.50	1005.75	0.98	0.02
CON034	384.21	404.58	488.50	364.88	370.08	407.00	346.71	338.92	372.50	867.75	0.85	0.00
CON035	332.29	348.08	391.00	309.25	346.42	370.50	313.58	351.42	332.00	788.34	1.00	0.00
CON036	420.71	443.58	473.00	394.71	418.83	419.00	405.96	378.08	382.00	960.52	1.00	0.00
CON037	416.13	445.25	544.00	417.67	430.75	407.50	404.25	374.42	449.00	786.64	1.00	0.00
CON038	416.79	451.00	491.00	377.29	500.50	460.00	421.33	373.33	338.50	811.74	0.94	0.06
CON039	355.71	382.17	423.50	312.42	358.92	401.50	334.13	320.83	399.50	723.42	0.98	0.02
CON040	369.21	379.50	437.50	345.96	385.42	404.00	363.13	373.42	395.50	940.53	0.98	0.02
CON041	391.25	420.83	505.00	374.33	399.42	404.00	389.29	409.83	375.50			

CON042			
CON043	836.64	0.96	0.00
CON044	968.51	0.96	0.00

Note: CCH group: from CCH001 to CCH032; Control group: from CON001 to CON044.
